# Potential impact of social media and COVID-19 restrictions on adult attention-deficit rates

**DOI:** 10.1192/bjb.2025.10162

**Published:** 2026-06

**Authors:** Premal Shah, Marios Adamou, Alison Cape, Martina Carboni, Dietmar Hank, Raja Anindya Sekhar Mukherjee, Chandrarajan Shah, Michael Smith

**Affiliations:** 1 Lothian Adult Autism and ADHD Team, Royal Edinburgh Hospital, Edinburgh, UK; 2 School of Human and Health Sciences, University of Huddersfield, Huddersfield, UK; 3 Avon and Wiltshire Mental Health Partnership NHS Trust, Bath, UK; 4 South London and Maudsley Mental Health NHS Trust, London, UK; 5 Surrey and Borders Partnership NHS Foundation Trust, Redhill, UK; 6 East of Scotland Surgical Training, Edinburgh, UK; 7 Adult ADHD Service, Leeds and York Partnership NHS Foundation Trust, Leeds, UK

**Keywords:** Autism, attention-deficit hyperactivity disorders, mental health services, service development, health economics

## Abstract

**Aims and method:**

We aimed to quantify attention-deficit hyperactivity disorder (ADHD) and autism assessment requests, and explore correlations with public interest and COVID-19 restrictions. We collected data on referrals to adult ADHD or autism services, Google searches for ‘autism’ or ‘ADHD’, birth gender ratios, ADHD prescriptions in England and COVID-19 restriction measures in four countries.

**Results:**

ADHD assessment demand tripled from July 2020 to January 2023, with Google searches for ADHD rising in parallel. Autism referrals and searches saw smaller, similarly timed rises. Female referrals outstripped males. ADHD prescriptions rose particularly in those aged 30–34 years. Google searches for ADHD unexpectedly rose from July 2020 in four countries, correlating with sustained intensity of national COVID-19 restrictions.

**Clinical implications:**

Public interest may have driven demand for ADHD assessments, with COVID-19 restrictions encouraging social media use facilitated by easy electronic information access. The public has decided that ADHD is important, independent of professional views. It is now critical that a consensus is reached to determine who benefits most from an ADHD diagnosis and medication.

Adult attention-deficit hyperactivity disorder (ADHD) and autism are common (with a prevalence of 2–4% and 1%, respectively^1^), shorten life expectancy^2^ and increase the risk major mental disorders – around 20% attending adult mental health services potentially have ADHD/autism.^3^

## Background

Many already stretched British adult ADHD and autism assessment services^4^ have recently been unexpectedly overwhelmed.^5^ A rational response requires accurate data quantifying demand and identifying driving factors. Autism demand has already been partially quantified.^6^ There is limited data on ADHD demand (e.g. NHS England does not collect data centrally on ADHD), although informal indicators suggest a larger rise.^7–9^

Although the factors driving demand are unproven, there are at least two possible candidates – the influence of social media and resultant increased public interest, and the influence of COVID-19 pandemic restrictions. The first is suggested by direct experience: one of our authors working in primary care noted many seeking ADHD assessment had done so after using social media, particularly TikTok. The second is suggested as the unexpected increase seems to have occurred around the time of the COVID-19 pandemic.

## Study aims

The two primary aims of this study were as follows:

- To quantify adult ADHD and autism assessment referral rates between 2019 and 2023 across representative services. We also explored if ADHD medication prescribing rates changed in parallel.
- To examine whether changes in ADHD/autism referral rates were related to proxy measures of changes in public interest and/or COVID-19 restrictions. We also explored if increased public interest increased the proportion meeting the criteria for referral for assessment.

## Method

### ADHD and autism assessment referral rates

We analysed anonymised, routinely collected monthly referral data between October 2018 and April 2023 from six UK regional adult autism and six ADHD services covering the same geographical population in a part of Scotland and across areas in the South-East, South-West and North-East of England. For each service, we calculated the average monthly referral rate for 6-month aliquots, from the beginning of April to end of September, and beginning of October to end of March, normalised to per 100 000 people aged 18–65 years covered by that service. As referral rates were similar across condition-specific services, mean monthly referral rates and 95% confidence intervals for each time aliquot were calculated with XLStat version 2023.2.1414 for macOS 15.6 (Lumivero, Burlington, Massachusetts, USA; see https://www.xlstat.com/en/).

### British public interest in autism and ADHD

Data was obtained with the ‘Keywords Everywhere’ algorithm,^10^ which provided quantitative UK-specific Google searches for the terms ‘ADHD’ and ‘autism’. The average monthly searches over each 6-month aliquot were calculated and standardised to per 10 000 people aged 18–65 years, using population figures from the Office for National Statistics.^11^

### Exploring if increasing public interest through social media had a specific effect in identifying those with possible ADHD/autism

Our basic assumption was that the number of people who attended their general practitioner (GP) seeking an autism/ADHD assessment was a fraction of the Google searches. It is possible that increased social media presence, and the concomitant increase in public interest in ADHD, specifically improved identification of those with potential ADHD. If this were the case, a larger fraction of those searching the term ‘ADHD’ on the internet (assumed to be a measure of public interest) may be referred for an ADHD assessment. Therefore, changes in the ratio of Google searches to ADHD referral rates could be an indirect indicator of whether an increase in public interest identified more appropriate people for referral. A smaller ratio could suggest proportionately more people reaching referral criteria. No change in the ratio despite increasing referral rates would suggest awareness was generally raised without increasing specificity.

### Birth gender and age at referral

Only one service was able to provide data on birth gender and average age at referral for composite ADHD and autism referral data. A gender ratio indicator was calculated by subtracting the proportion of males from the proportion of females. A figure of 0 represents a male:female ratio of 1:1, −1 represents a ratio of 2:1 and +1 represents a ratio of 1:2.

### Prescribed ADHD medication

The number and age ranges of people in England receiving prescribed ADHD medication from 2015 to 2022 was extracted from freely available data from the NHS Business Authority.^12^

### International public interest in ADHD and stringency of governmental COVID-19 measures

As quantitative data was not available for all countries, relative interest search data from Google Trends^13^ for the search terms ‘ADHD’ for the UK, USA, Sweden and India over the past 5 years were used. Google Trends produces relative search figures (indicating relative interest) for a specified search term for the chosen country, assigning ‘100’ to the maximum number of searches in a week within the specified period, with the country’s other weekly figures being scaled accordingly. For each country, average relative interest for ‘ADHD’ over the 6-month aliquots described above were calculated.

We quantified increases in Google searches as:

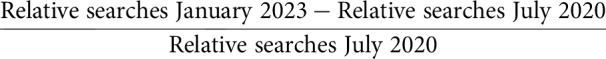




Publicly available daily Stringency Index data from the Oxford Coronavirus Government Response Tracker project^14^ were used. The daily Stringency Index quantifies a government’s COVID-19 stringency measures by day. The peak Stringency Index and the duration for which it was 50 or greater (50 chosen arbitrarily) were noted. From this, we formed a new measure – the Average Sustained Intensity of COVID-19 Measures (ASIM50), calculated as:

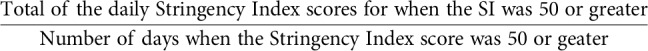




The greater the ASIM50, the more intense and sustained were the average COVID-19 restrictions. ASIM50s were calculated for the UK, USA, Sweden and India. These countries were chosen because of differing government COVID-19 pandemic measures, and because they allowed access to TikTok during the study period.

## Results

### Referral rates

The sampled services covered around 10% of the British population (approximately 6.7 million people). Two services provided composite ADHD and autism referral data, and four provided separate ADHD and autism referral data.

**Fig. 1:**
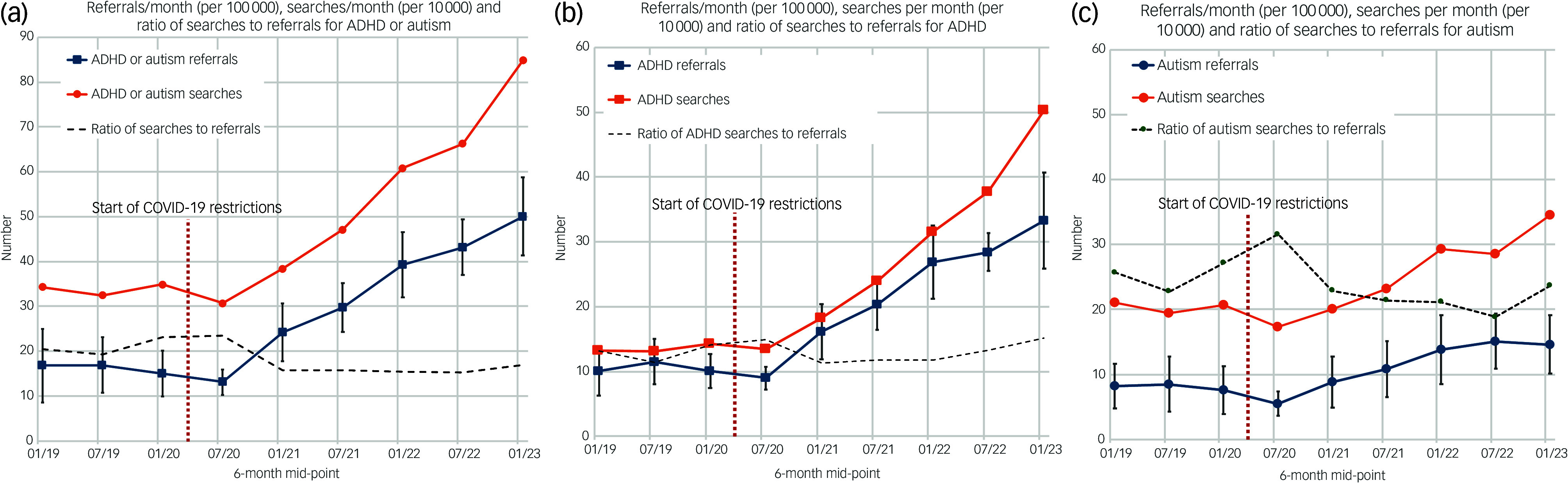
(a) ADHD or autism assessment referrals across six regional services, Google searches and ratio of searches to referrals. (b) ADHD referrals across four regional ADHD services, Google searches and ratio of searches to referrals. (c) Autism referrals across four regional autism services, Google searches and ratio of searches to referrals. Error bars represent +/− 2 s.e.m. ADHD, attention-deficit hyperactivity disorder.

All services experienced unexpected increases from July 2020 (Fig. 1). From July 2020 to January 2023, combined ADHD and autism services saw a 2.9 times increase in referrals, from 17 to 50 referrals per 100 000 people per month. This was mainly attributable to increased ADHD referrals, which tripled from 11 to 33 per 100 000 people per month, an average increase of 80% per annum. In comparison, autism referrals increased 1.72 times, from 8.5 to 14.6 per 100 000 people per month.

### UK public interest in ADHD and autism

Google searches for ‘ADHD’ or ‘autism’ increased unexpectedly from July 2020, increasing 2.6 times (from 33 to 85 per month per 10 000 people) from July 2020 to January 2023 (Fig. 1). ‘ADHD’ searches increased 3.8 times, from 13 to 50 searches per month per 10 000 people. Both ADHD searches and referrals increased unexpectedly from July 2020, and the size of increase for both was similar. A similar pattern, but smaller increases, were observed for autism.

### Ratio of internet searches to referrals

The ratio of ADHD searches to referrals was 13:1, remaining unchanged over 4 years (Fig. 1). The ratio of autism searches to referrals was around 23:1, also remaining unchanged.

### Birth gender and age of those referred

**Fig. 2:**
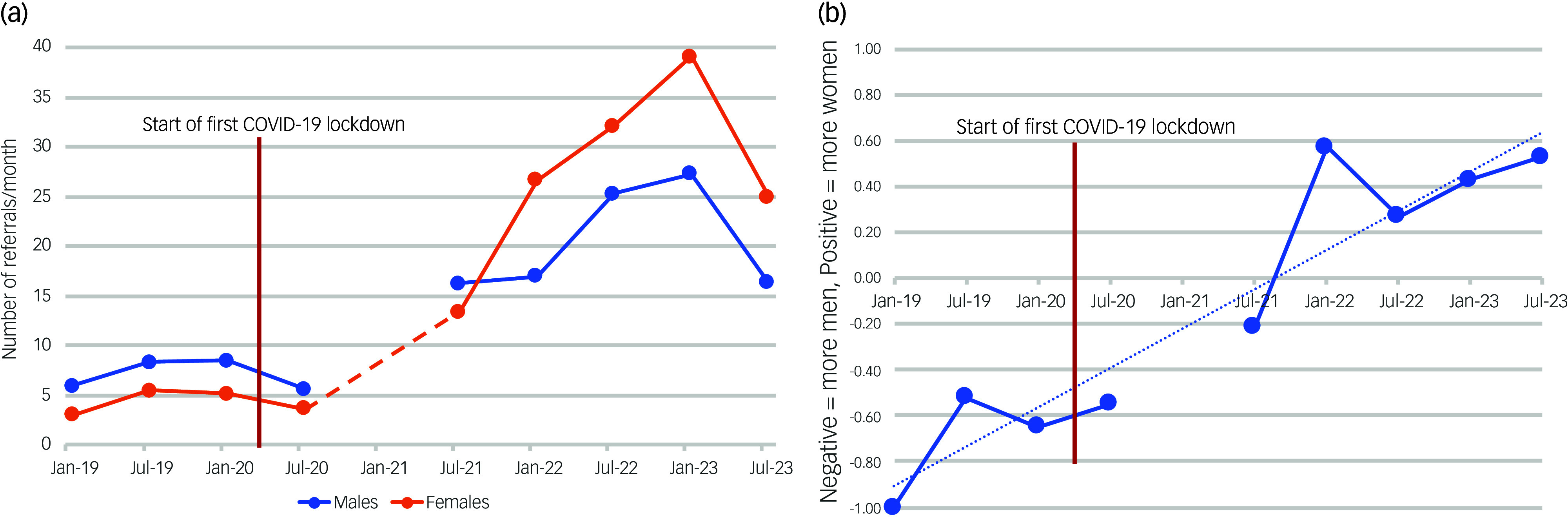
(a) Number of referrals for ADHD or autism assessment by gender from January 2019 to July 2023. (b) Birth gender ratio indicator for ADHD and autism referrals between January 2019 and July 2023. An indicator of −1 equates to a male:female ratio of 2:1, 0 equates to a ratio of 1:1 and 1 equates to a ratio of 1:2. ADHD, attention-deficit hyperactivity disorder.

One service provided birth gender data and age of those referred for ADHD or autism assessment. Pre-pandemic, more males than females were referred. The male:female ratio gradually switched from January 2019 (2:1) to July 2023 (1:1.5) (Fig. 2). There was no change in the average age of those being referred before July 2020 (mean 31.5 years) compared with after July 2020 (mean 31.4 years).

### ADHD prescribing data

**Fig. 3:**
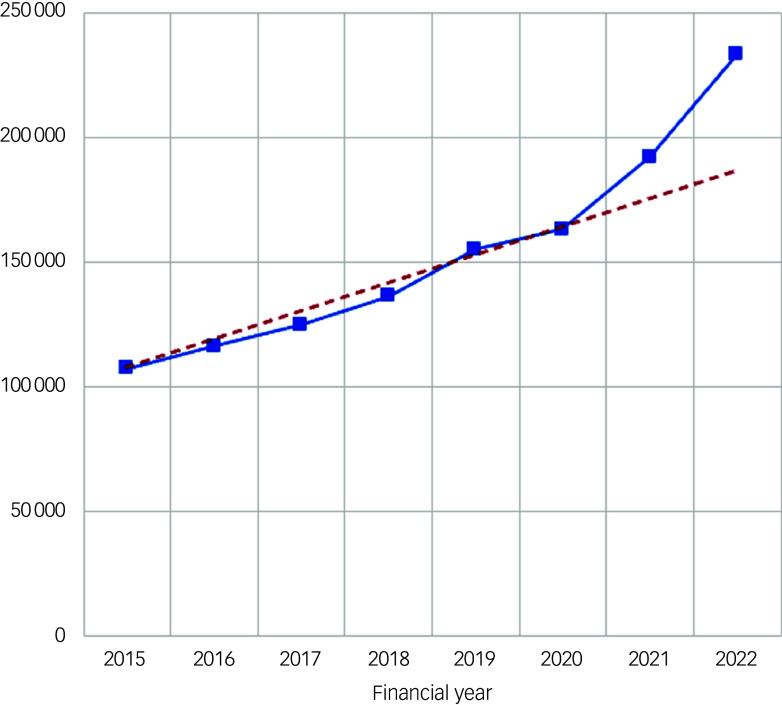
Number of people receiving prescriptions for ADHD medication issued in England by year. ADHD, attention-deficit hyperactivity disorder.

The average annual rate of increase in those prescribed ADHD medication doubled after 2020 (Fig. 3). Before 2020, prescriptions rose by 10.4% per annum, whereas after 2020, prescriptions rose by 21% per annum. ADHD referral rates remained relatively static before 2020, but rose by 80% per annum after 2020.

Post-COVID-19, there was a 146% increase in prescriptions in the 30–34 year age group. The 25–44 year age group correlates to the largest users of TikTok,^15^ encompassing 49.8% of users.

### Internet searches for ‘ADHD’ and COVID-19 stringency measures for four countries

**Fig. 4:**
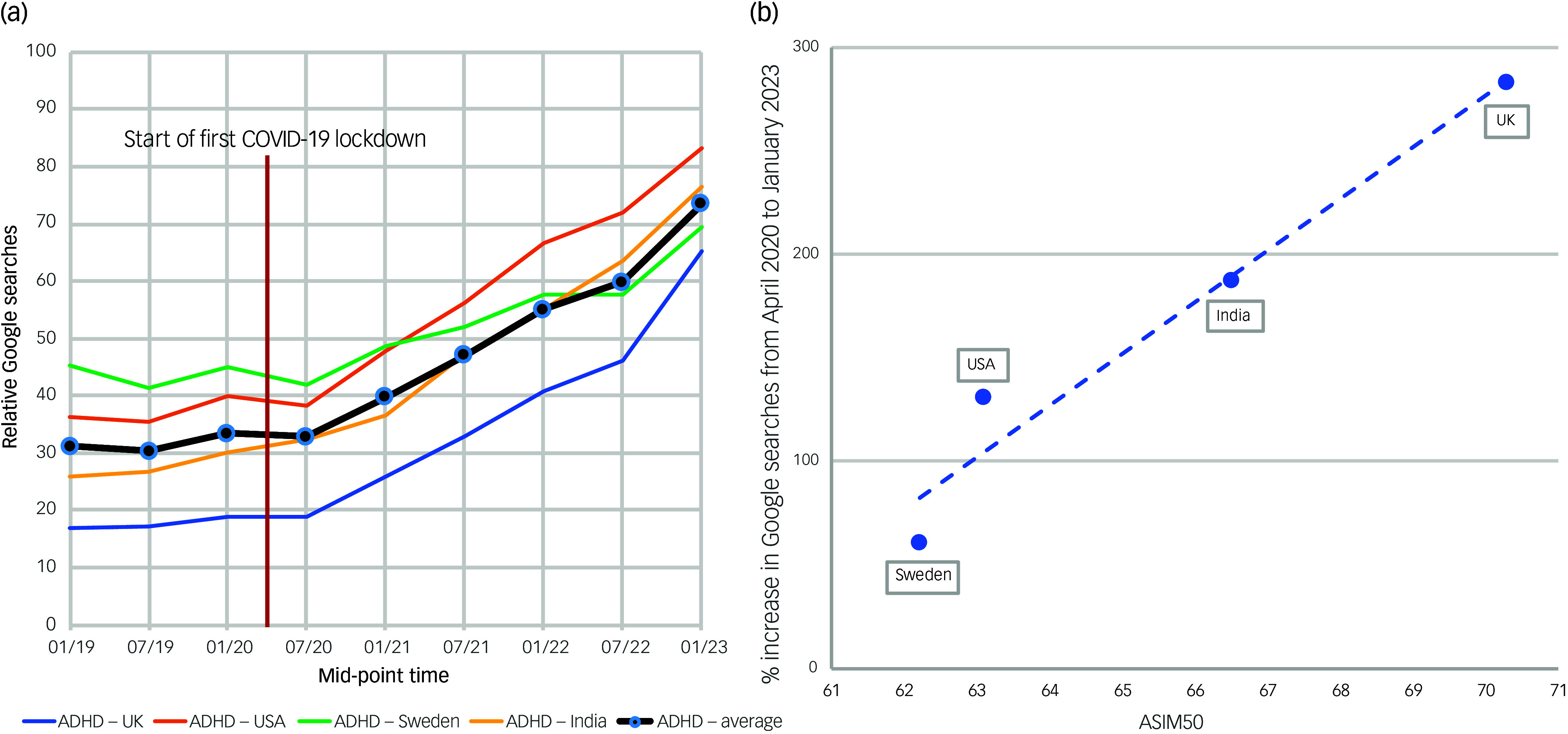
(a) Relative Google searches for ADHD for four countries. (b) Relationship between size of increase in Google searches and ASIM50. ADHD, attention-deficit hyperactivity disorder; ASIM50, Average Sustained Intensity of COVID-19 Measures.

All four countries experienced unexpected increases in ADHD searches from July 2020, with an average increase of 2.4 times between July 2020 and January 2023 (range 1.7–3.8 times) (Fig. 4).

All four countries imposed restrictions greater than 50 on the Stringency Index in the second to third week of March 2020. The UK had the highest ASIM50 and Sweden had the lowest (Table 1).

**Table 1:** Comparison of COVID-19 stringency measures in four countries

Country	OCGRT peak Stringency Index	ASIM50	Duration of ASIM50 (days)
UK	88	70.3	484
India	100	66.5	764
USA	75.5	63.1	624
Sweden	71.1	62.2	473

OCGRT, Oxford Coronavirus Government Response Tracker project; ASIM50, Average Sustained Intensity of COVID-19 Measures.

Increasing ASIM50 was associated with a greater increase in searches for ADHD. Neither the duration of the ASIM50, nor the peak Stringency Index, were associated with the magnitude of increase in searches.

## Discussion

### Findings

To the best of our knowledge, this paper is the first to evidence that from mid-2020, increased social media/public interest and COVID-19 restrictions may have been associated with the unexpected increased demand for ADHD assessments (and, to a smaller extent, with autism), independent of service parameters.

### Indirect indicators

There were several indirect indicators that demand for ADHD assessment had risen: many clinicians from regional adult autism and ADHD services (within the purview of the Neurodevelopmental Disorders Special Interest Group of the Royal College of Psychiatrists) had reported unexpected increases in ADHD referrals exceeding capacity;^16^ service saturation had caused some NHS services^17^ and ‘Right to Choose partners’^7^ to either pause their ADHD waiting lists, prioritise high-risk individuals (e.g. Jayanetti^8^) or quote lengthening waiting times; the ADHD Foundation (a neurodiversity charity providing pre-diagnostic screening) quoted a 400% increase in demand for ADHD assessment since 2020;^7^ ADHD assessment in the UK private sector had recently expanded inordinately;^17^ NHS England responded by forming an ADHD Programme Clinical Reference Group^17^ and the worldwide shortage of ADHD medication^12,18^ suggested that diagnosis and demand had increased internationally.

### Current data

ADHD assessment demand was greater than that for autism from the beginning of the sample period (January 2019), and accelerated disproportionately from mid-2020. Average ADHD referral rates rose unexpectedly by a factor of 3, from 11 to 33 per 100 000 people per month, matching increases in public interest (factor of 3.8). Prescriptions also rose unexpectedly after 2020, possibly representing the delayed effects of increased ADHD referrals and diagnosis, with the extent of the rise plausibly limited by service capacity. Preliminary data also suggests a significant shift from predominantly males to females being referred, which is unexpected given the historically accepted gender distributions for ADHD and autism. This started pre-pandemic, changing gradually between 2019 and 2023. This shift parallels recent prescribing data showing a marked rise in ADHD medication prescriptions in females.^12^ It was observed that the greater the degree of sustained COVID-19 restrictions (ASIM50), the greater the increase in the public’s interest in ADHD, with the UK being at the extremes for both.

TikTok’s use increased markedly during 2020, either coincidentally or as an unforeseen consequence of COVID-19. TikTok was the most downloaded non-gaming application in 2021 and 2022,^19^ with downloads peaking in the first half of 2020. ADHD was ‘massive on TikTok’,^20^ and the tag #ADHD was viewed around 1.19 billion times a month from June 2022 to November 2023^21^ – equivalent to one in five of the world’s adults viewing an ADHD video every month.

It was not possible to freely obtain data on the number of active TikTok users or the numbers exposed to ADHD on TikTok by year and country. However, this may not be the most useful or accurate metric: unlike other social media, TikTok serves content that may not be directly related to the person’s interests.^22^ Because of this, a better measure of sustained public interest may be internet searches initiated by individuals themselves. Therefore, Google searches were used as the proxy measure of public interest, because Google is the most frequently used internet search engine worldwide.

The COVID-19 pandemic was the most prominent event of 2020. Although COVID-19 has been associated with subsequent neuropsychiatric symptoms,^23^ these are unlikely to represent ADHD, which is characterised by lifelong traits causing significant dysfunction and would predate COVID-19. It is more likely that referral rates were influenced by indirect or coincidental factors such as COVID-19 social restrictions. To assess this, we derived the ASIM50 as a proxy measure of sustained intensity of restrictions applied to a country. As it was not possible to obtain reliable referral data for other countries, the ASIM50 was correlated with Google search data, because of its possible correlation with referral rates. ADHD searches increased across all four countries from mid-2020, with a correlation between the ASIM50 and the magnitude of increase in ADHD searches.

### Possible interpretation

It seems that several partially independent events interacted around the time of the COVID-19 pandemic, to lead to the large unpredicted increase in ADHD assessment. First, we know that adult ADHD is significantly underidentified in the UK, particularly in females.^2^ Second, like most of the world, the UK population underwent significant COVID-19 restrictions from early 2020. Unlike past pandemics, many people had internet access facilitating the use of electronic media for social contact and general information. Third, perhaps coincidentally or because of lockdown restrictions, TikTok’s use ‘exploded’ around this time. TikTok’s unique algorithm potentially exposed the public to material that they would not necessarily seek out, potentially leading to a worldwide audience watching videos labelled as ADHD, without ADHD being their primary interest. This may have led to an increase in awareness of ADHD, including ADHD in females. This then led to more people seeking information through internet searches, which then led to more people seeking assessment. Our data suggests that this had the effect of increasing general awareness: it did not change the ratio of searches to referrals.

The possible correlation between the ASIM50 and internet searches could also suggest that more severe sustained restrictions were associated with greater use of electronic media for social purposes and resultant greater exposure to ADHD-labelled material. A possible implication is that demand for adult ADHD assessments increased in other countries proportionate to the increase in public interest, although this remains to be proven. It is possible that lockdown measures had a disproportionately negative impact on those with unidentified ADHD; however, it is not possible to conclude this with certainty based on our findings.

Several factors are therefore suggested to have coincided to lead to the unprecedented rise in ADHD referrals: pre-existent diagnostic underidentification, social restrictions leading to an in social media use, TikTok’s unique algorithm increasing general awareness and easy access to further information.

### Social media and mental health conditions

The effects of TikTok and other social media on raising awareness and identification of health conditions, and their impact on services, is yet to be determined. Preliminary evidence^24^ suggests that younger adults may be turning to social media to self-diagnose or self-identify, with the main perceived benefit being validation and support.^25^ However, social media’s diagnostic accuracy is highly variable. Yeung et al^26^ found that around half of sampled ‘ADHD’ TikTok videos were misleading, and Olvera et al^27^ found the depictions of tics on TikTok differed significantly from typical tic disorders. This raises legitimate concerns that increased assessment demand may be driven by those not meeting screening criteria. However, our data do not support this view, but suggest that social media had a general awareness-raising effect.

### Limitations

The participating services encompass around 10% of the British population, from heterogeneous socioeconomic and demographic backgrounds, and service configurations. Despite this, service referral rates and patterns of change were sufficiently similar to allow use of parametric summary statistics. Further, the size and heterogeneity of the sample make these findings generally applicable.

This study necessarily relies heavily on derived or proxy measures of public interest, social media use and social restriction. These findings also establish associations rather than causality. It is important to recognise the limitations of derived and correlative data. Despite these limitations, the data supports the idea that social media has had a significant and lasting effect on ADHD referral rates beginning early during the COVID-19 pandemic. This hypothesis is strengthened by the observation of an independent, smaller, but similar effect on autism referral demand.

### Implications

Public interest, however generated, can drive demand, which is the essence of successful advertising. Our findings suggest that social media and easy access to information may have increased public interest and individuals’ awareness of specific health issues, possibly leading to significant ‘knock-on’ effects for healthcare demand, as appears to be the case with ADHD.

The current findings are novel for several reasons. They suggest a significant ‘bottom-up’ drive in demand, with the public identifying ADHD as an important health concern independent of professionals’ views. They illustrate how rapidly, unpredictably and profoundly health service demand can increase given a change in circumstances, ready access to social media and easily available information. However, we do not know if these effects are predictable or easily modifiable.

These are important considerations because there is a sharp focus on commissioning demands for ADHD services. Understandably, some services have responded with short-term measures prioritising those perceived to be at greatest clinical risk, as no service can absorb an unplanned 300% increase in demand. Although several options have already been described,^16^ stakeholders now need to collaborate to determine who benefits most from a medical diagnosis in dimensional disorders, who benefits most from a psychiatric/psychological approach to ADHD and who benefits most from medication.

The lower cut-off for each of these is unknown, with matters being complicated by the absence of an agreed severity scale for both autism and ADHD. The answers to these are the cornerstones of a logical strategic response based on ‘realistic medicine’,^28^ ensuring the optimal use of health service time, expertise and resources.

## Data Availability

The relevant aggregated data and internet search data can be made available on reasonable request to the corresponding author, P.S.

## About the authors

**Premal Shah**, BSc, MBChB, MD, FRCPsych, is a consultant psychiatrist with the Lothian Adult ADHD and Autism Team at Royal Edinburgh Hospital, NHS Lothian, Edinburgh, UK. **Marios Adamou**, PhD, MD, FRCPsych, is a consultant psychiatrist with South West Yorkshire Partnership NHS Foundation Trust, Wakefield, UK; and a professor at the School of Human and Health Sciences, University of Huddersfield, Huddersfield, UK. **Alison Cape**, MBChB, MRCPsych, is a consultant psychiatrist with Avon & Wiltshire Mental Health Partnership NHS Trust, Bath, UK. **Martina Carboni**, MBBS, Specialty Diploma in Psychiatry, MD, is a consultant psychiatrist with South London and Maudsley NHS Foundation Trust, London, UK. **Dietmar Hank**, MSc, PgDpl (neuropsychiatry), MRCPsych, is a consultant psychiatrist with Avon & Wiltshire Mental Health Partnership NHS Trust, Bath, UK. **Raja Anindya Sekhar Mukherjee**, MBBS, PhD, FRCPsych, is a consultant psychiatrist with Surrey and Borders NHS Partnership, Redhill, UK; and Honorary Professor at the University of Salford, Salford, UK. **Chandrarajan Shah**, BSc, MBChB, MRCS, is a core surgical trainee with East of Scotland Surgical Training, Edinburgh, UK. **Michael Smith**, MSc, MBChB, MRCPsych, is a consultant psychiatrist with Leeds and York Partnership NHS Foundation Trust, Leeds, UK.
